# Serum Metabolomics Analysis in Wasp Sting Patients

**DOI:** 10.1155/2018/5631372

**Published:** 2018-12-25

**Authors:** Xianyi Yang, Lin Chai, Chunyan Liu, Mei Liu, Limei Han, Changsheng Li, Hui Guo, Yuwen Sun, Xiaomin Rao, Min Xiao, Zhicheng Fang

**Affiliations:** ^1^Emergency Department, Taihe Hospital, Shiyan 442000, China; ^2^Department of Pediatrics, Shiyan Maternal and Child Health-Care Hospital, Shiyan 442000, China; ^3^Department of Pediatrics, Taihe Hospital, Shiyan 442000, China

## Abstract

To analyze the dynamic changes of serum metabolomics in wasp sting victims, we collected serum from 10 healthy volunteers and 10 patients who had been stung 3 hours, 24 hours, and 72 hours before sample collection. We analyzed the metabolomics by high-performance liquid chromatography-tandem mass spectrometry (HPLC-MS/MS) techniques and then performed enrichment analysis. A total of 838 metabolites were identified. Serum metabolomics analysis using MetaboAnalyst revealed 289 metabolites that were significantly different among patients in the 3-hour group versus healthy controls (P<0.001). Pathway analysis of those metabolites indicated that those metabolic sets were associated with sphingolipid metabolism. Based on the differences among the control, 3-hour, 24-hour, and 72-hour groups, we classified serum metabolites into different categories. The first and second categories included 297 and 280 metabolites that were significantly different in terms of concentration among healthy controls versus the participants whose sera were analyzed 3 hours, 24 hours, and 72 hours after wasp stings. Pathway analysis of those metabolites indicated that those metabolic sets were associated with thiamine metabolism. The third category included 269 significant metabolites. The fourth category included 28 significant metabolites. Pathway analysis of the metabolites in third and fourth categories indicated that those metabolic sets were associated with phenylalanine, tyrosine, and tryptophan biosynthesis. The fifth category included 31 metabolites, which were not significantly different between the control and 3-hour groups but were higher in concentration in the 24-hour and 72-hour groups. Pathway analysis of the fifth category of significant metabolites identified linoleic acid metabolism. In conclusion, multiple metabolic pathways are associated with wasp stings, and these might provide a basis for exploring mechanisms of wasp sting injury and potential targets for therapy.

## 1. Introduction

Wasps belong to the order Hymenoptera, suborder Apocrita, and superfamily Vespoidea. There are more than 6000 kinds of wasps in the world and more than 200 kinds of wasps in China [1]. Wasps are widely distributed, with different compositions of wasp venom in different genera [2]. Anaphylaxis caused by wasp venom can often result in personal injury and death, with the incidence of morbidity increasing, especially in industrialized countries [3]. Wasp stings can cause allergic and toxic reactions, which in severe cases can lead to multiple organ dysfunction. Acute renal injury is often the most prominent form of organ dysfunction; high creatinine levels, shock, oliguria, and anemia are risk factors for death among wasp sting patients [4].

Wasp venom is known as a potent natural drug, with anti-inflammatory, bactericidal, antiviral, and antitumor effects [5]. It can also be used clinically for the treatment of multiple sclerosis, rheumatoid arthritis, and Parkinson's disease. Nevertheless, the target of wasp venom and the mechanisms of allergic reaction remain unclear.

The development of modern molecular biological techniques has improved our understanding of wasp venom components. The amine components are serotonin, histamine, dopamine, and noradrenaline, which constitute the bulk of the small molecules in wasp venom [6]. The peptide components are melittin, apamin, mast cell degranulation peptides, antibacterial peptides, bradykinin, and chemotactic peptides [7–14]. Protein electrophoresis of wasp venom has demonstrated high protein concentrations at 23, 34, and 43 kD, sites identified as three major protein components: antigen-5 protein, phospholipase A1, and hyaluronidase [15–18].

Serum peptides can be used to distinguish a normal, healthy individual from someone who has recently been stung by a wasp [19]. After analyzing the serum levels of 34 amino acids, Matysiak et al. demonstrated that the levels of L-glutamine (Gln), L-glutamic acid (Glu), L-methionine (Met), and 3-methyl-L-histidine (3MHis) were significantly different between wasp sting victims and healthy controls [20]. Alonezi et al. studied the effect of one major component of wasp venom, apamin, on the metabolism of ovarian cancer cells and showed that apamin regulates the levels of metabolites during the tricarboxylic acid (TCA) cycle, oxidative phosphorylation, purine and pyrimidine metabolism, and the arginine/proline pathways [21, 22]. Using nuclear magnetic resonance (NMR) techniques, Zhao et al. detected serum metabolite changes in rats treated with wasp venom and found 14 metabolites that were significantly different between normal controls and rats that had received wasp venom 3 hours before serum analysis [23]. However, due to the low sensitivity of NMR technology, many metabolites are not identified [23]. Serum metabolite patterns in humans after wasp stings have not yet been comprehensively studied.

A holistic understating of complex biological systems by metabolomics can reveal endogenous metabolites and metabolism pathways during the development and treatment of diseases and provide a mechanistic basis for overall body function and drug action [21, 22]. This project aimed to use high-performance liquid chromatography-tandem mass spectrometry (HPLC-MS/MS) to detect sequential human serum metabolite concentration changes associated with wasp stings.

## 2. Materials and Methods

### 2.1. Patients

Ten wasp sting patients (7 males and 3 females stung by the same species of wasp—the common wasp—with a median age of 35.73±18.26) and 10 healthy controls were enrolled between July 2015 and October 2017 from Taihe Hospital, China. Sera were collected at 3 hours, 24 hours, and 72 hours after the wasp stings. Sera from 2 patients were mixed in equal volumes before tests. This study was approved by the Ethics Committee of Taihe Hospital, China. All patients provided written informed consent before participating.

### 2.2. Sample Preparation

All the serum samples were thawed at 4°C before analysis; 200 *μ*l of serum was mixed with 400 *μ*l of methanol (Merck, Germany), vortexed for 30 seconds, stored for 2 hours at −20°C, and then centrifuged at 13,000 rpm for 15 minutes at 4°C. The supernatant was mixed with 200 *μ*l of 50% methanol, vortexed for 30 seconds, centrifuged at 13,000 rpm for 15 minutes at 4°C, and filtered with a 0.22 *μ*m filter. To make a quality control sample, 20 *μ*l supernatants from each sample were mixed.

### 2.3. High-Performance Liquid Chromatography-Tandem Mass Spectrometry (HPLC-MS/MS)

Samples were analyzed by using AB 5600+ Triple TOF mass spectrometry (AB Sciex, USA) with an electrospray ionization (ESI) source attached to an Ekspert UlatraLC 110 system (AB Sciex, USA). Serum samples were separated using an ACQUITY UPLC HSS T3 column (1.8 *μ*m, 2.1 mm × 100 mm, Waters, USA) and monitored with mobile phases A (water:acetonitrile:formic acid, 900:100:1) and B (acetonitrile:water:formic acid, 900:100:1). The gradient elution at 0–4 minutes: 0% B, 4–6 minutes: 25% B, 6–29.1 min: 100% B, and 31–33 min: 0% B. The separation from the HPLC series was directly sprayed into the mass spectrometer. All mass spectrometry data acquisition was performed based on IDA function using the control software Analyst TF 1.7 (AB Sciex) with a scanning range of m/z 50–1000 for primary and m/z 25–1000 for secondary, and the secondary bombardment energy was 30 eV. The parameters of the ESI ion source were set as follows: atomization pressure (GS1): 55 Pa, auxiliary air pressure: 55 Pa, air curtain pressure: 40 Pa, temperature: 55°C, spray voltage: 5500 V, and positive ion mode.

### 2.4. Metabolomics Statistical Analysis

Raw data were converted to.abf format, using Analysis Base File Converter software, and then imported into MSDIAL2.76 software for data processing, such as peak search, peak alignment, and normalization. Matrix containing information, such as substance name, retention time, and charge-to-mass ratio was obtained by searching the MassBank, MoNA, Respect, and GNPS databases. The dataset was analyzed using MetaboAnalyst 3.6 (http://www.metaboanalyst.ca/faces/home.xhtml) after removal of the missing values (CV<30% of quality control samples) [21]. Using supervised analysis, such as partial least-squares discriminant analysis (PLS-DA), the different variances were aggregated. Significant metabolites (fold changes >2, P<0.05) were matched to metabolomics pathways using the pathway analysis and enrichment analysis (KEGG) features in MetaboAnalyst 3.6 [22]. Differences among the control, 3-hour, 24-hour, and 72-hour groups were analyzed using Prism 7.0 (GraphPad software, USA) with two-tailed t-tests followed by one-way ANOVA. P<0.05 was considered statistically significant.

## 3. Results

### 3.1. Serum Metabolome Alterations in Wasp Sting Patients

**Figure 1:**
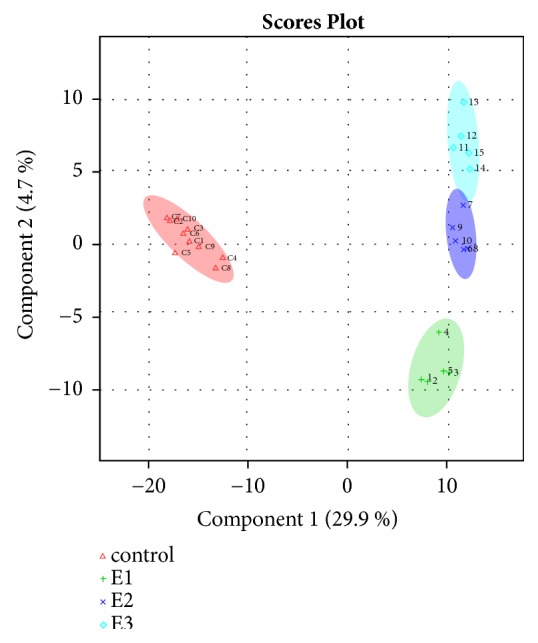
**Partial least squares discriminant analysis (PLS-DA) of metabolites from healthy controls and patients 3 hours, 24 hours, and 72 hours after wasp stings**. N=10 biological replicates/group. E1: 3-hour group; E2: 24-hour group; E3: 72-hour group after wasp sting.

A total of 838 metabolites were identified. PLS-DA analysis of the results identified a clear separation between the control group and the wasp sting (3-hour, 24-hour, and 72-hour) groups (Principal Component 1, 29.9%), as well as between the 3-hour, 24-hour, and 72-hour groups along Principal Component 2 (4.7%) (Figure 1).

**Figure 2:**
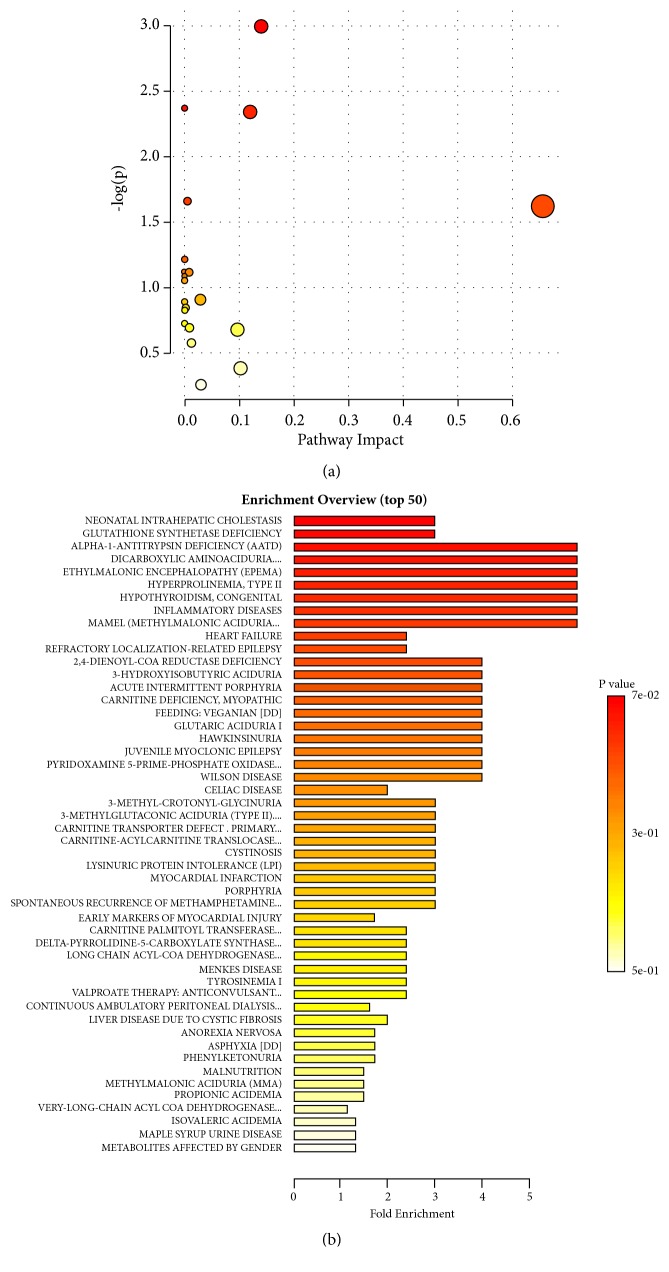
**Metabolomics analysis of serum from healthy controls and the 3-hour wasp sting group**. (a) Pathway analysis of significant metabolites. (b) Pathway enrichment of significant metabolites using metabolic datasets. a. Sphingolipid metabolism, P<0.05.

There were 289 metabolites that were significantly different between healthy controls and individuals in the 3-hour group, with 204 decreased and 85 increased in the 3-hour group. Pathway analysis of the 289 significant metabolites identified multiple pathways—including sphingolipid metabolism—with P values <0.05 (Figure 2(a)). Pathway enrichment analysis using metabolic sets identified neonatal intrahepatic cholestasis, glutathione synthetase deficiency, alpha-1-antitrypsin deficiency, dicarboxylic aminoaciduria, hyperprolinemia type II, hypothyroidism, and inflammatory diseases with P values <0.05 (Figure 2(b)).

### 3.2. The Dynamic Changes of Serum Metabolites among the 3-Hour, 24-Hour, and 72-Hour Wasp Sting Patients

Based on the differences among the control, 3-hour, 24-hour, and 72-hour groups, we classified the serum metabolites into 6 categories.

**Figure 3:**
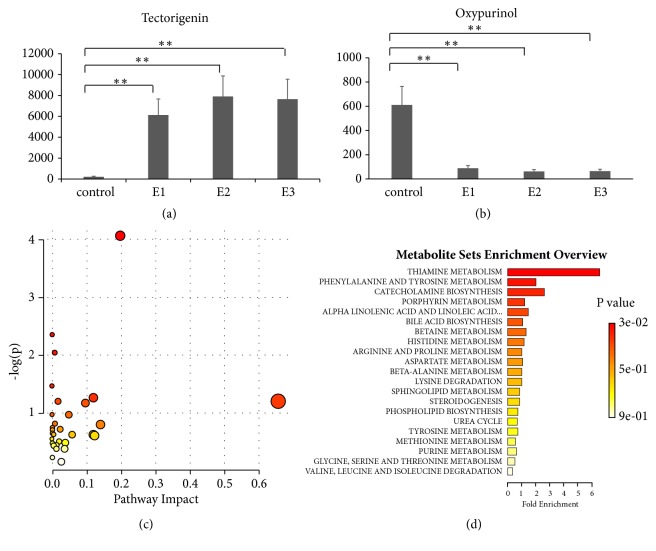
**Dynamic changes of serum metabolites**. (a) Representative metabolites in the first category. (b) Representative metabolites in the second category. (c) Pathway analysis of significant metabolites in the first and second categories. (d) Pathway enrichment of significant metabolites in the first and second categories. E1: 3-hour group; E2: 24-hour group; E3: 72-hour group after wasp sting. a. Thiamine metabolism, P=0.017.

**Figure 4:**
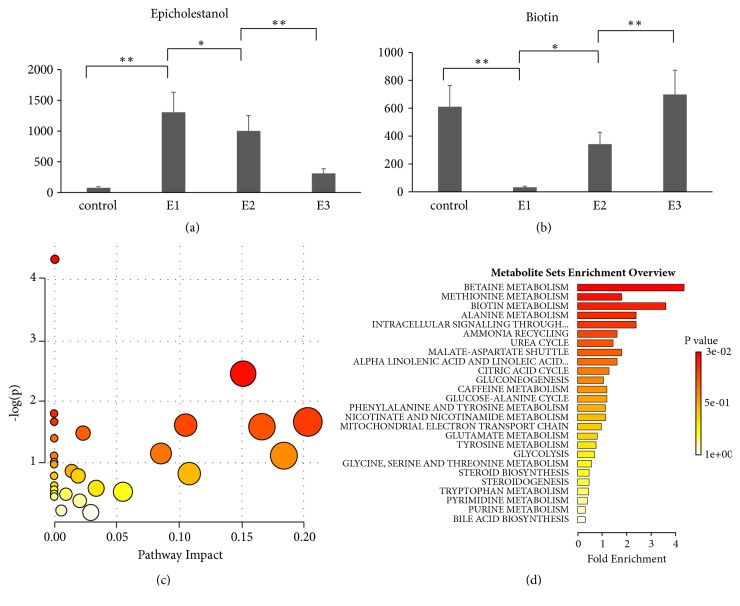
**Dynamic changes of serum metabolites**. (a) Representative metabolites in the third category. (b) Representative metabolites in the fourth category. (c) Pathway analysis of significant metabolites in the third and fourth categories. (d) Pathway enrichment of significant metabolites in the third and fourth categories. E1: 3-hour group; E2: 24-hour group; E3: 72-h group after wasp sting. a. Phenylalanine, tyrosine, and tryptophan biosynthesis, P=0.013.

The first category included 297 metabolites that were of lower concentration in the control group than in the 3-hour, 24-hour, and 72-hour groups (e.g., tectorigenin, Figure 3(a)). The second category included 280 metabolites that were of higher concentration in the control group than in the 3-hour, 24-hour, and 72-hour groups (e.g., oxypurinol, Figure 3(b)). Pathway analysis of these 577 significant metabolites identified multiple pathways, including thiamine metabolism (P=0.017) (Figure 3(c)). Pathway enrichment analysis using metabolic sets identified thiamine metabolism, phenylalanine and tyrosine metabolism, and catecholamine biosynthesis with P values <0.05 (Figure 4(d)).

The third category included 269 metabolites (3-hour group > 24-hour group > 72-hour group, in terms of serum concentration, all of which were not significantly different with the control group). For example, epicholestanol (Figure 4(a)) was one of the metabolites in the third category. The fourth category included 28 metabolites (3-hour group < 24-hour group < 72-hour group, in terms of serum concentration, all of which were not significantly different with the control). Biotin (Figure 4(b)) was in the fourth category. Pathway analysis of these significant metabolites identified multiple pathways—including phenylalanine, tyrosine, and tryptophan biosynthesis (P=0.013) (Figure 4(c)). Pathway enrichment analysis using metabolic sets identified betaine metabolism, methionine metabolism, biotin metabolism, alanine metabolism, and intracellular signaling through prostacyclin receptor and prostacyclin, all as having P values <0.05 (Figure 4(b)).

**Figure 5:**
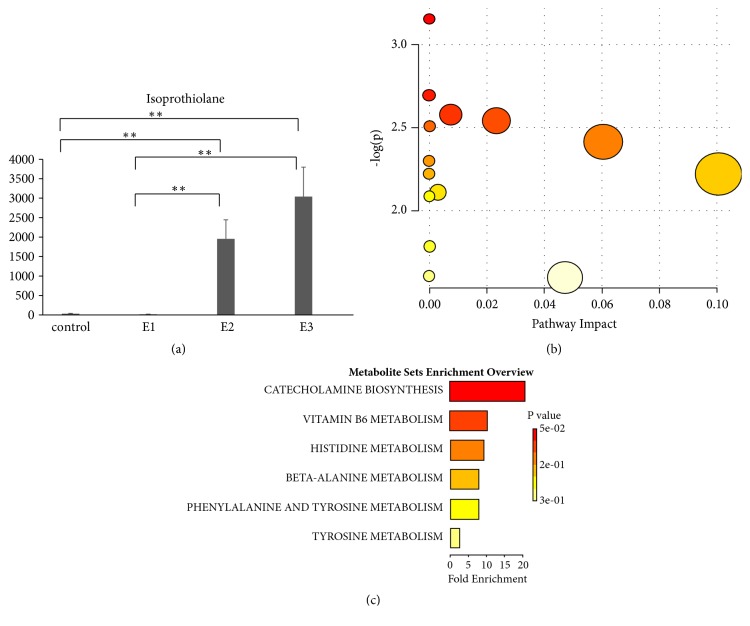
**Dynamic changes of serum metabolites**. (a) Representative metabolites in the fifth category. (b) Pathway analysis of significant metabolites in the fifth category. (c) Pathway enrichment of significant metabolites in the fifth category. E1: 3-hour group; E2: 24-hour group; E3: 72-hour group after wasp sting. a. Linoleic acid metabolism, P<0.05.

The fifth category included 31 metabolites, which were not significantly different between the control and 3-hour groups but were higher in concentration in the 24-hour and 72-hour groups. Isoprothiolane (Figure 5(a)) is an example from the fifth category. Pathway analysis of these significant metabolites identified multiple pathways—including linoleic acid metabolism—with P values <0.05 (Figure 5(b)). Pathway enrichment analysis using metabolic sets identified catecholamine biosynthesis, vitamin B6 metabolism, and histidine metabolism as having P values <0.05 (Figure 5(c)).

The sixth category induced other metabolites.

## 4. Discussion

Wasp venom contains allergens and toxins, which could induce allergic and toxic reactions. Anaphylaxis is easy to cure, but toxic reactions can lead to acute renal failure, liver failure, multiple organ failure, and death. The analysis of serum metabolism after wasp stings might provide new ideas for understating the pathogenic mechanisms of wasp stings and a theoretical basis for clinicians to minimize multiple organ failure and death.

In this study, HPLC-MS was used to study the dynamic changes of metabolism in the peripheral blood of patients after wasp stings. We identified a total of 838 metabolites. PLS-DA analysis of these metabolites identified a clear separation between the control group and the wasp sting (3-hour, 24-hour, and 72-hour) groups (Principal Component 1, 29.9%), as well as between the 3-hour, 24-hour, and 72-hour groups along Principal Component 2 (4.7%).

Matysiak et al. performed nontargeted metabolome assays with the aTRAQ method and found that Glu was upregulated, but the Gln and histidine (3MHis) amino acid concentrations did not change significantly after wasp stings [19]. Our data show that Glu was upregulated, while other amino acids were not detected. This may be due to the sensitivity of the instruments. Matysiak et al. used aTRAQ methodology [19], and the nontargeted metabolome assays used in our study had a lower sensitivity. Both pathway analysis and pathway enrichment analysis revealed tyrosine metabolism as involving borderline nonsignificant metabolites. This is consistent with the findings of Zhao et al. who directly stimulated rats with wasp venom, observed the dynamic changes of serum metabolites, and found that the metabolites were closely associated with methane, glycine, dicarboxylate, arginine, and proline metabolism as well as the TCA cycle [23]. In this study, there were other significant pathways detected, including those involved in neonatal intrahepatic cholestasis, glutathione synthetase deficiency, alpha-1-antitrypsin deficiency, dicarboxylic aminoaciduria, hyperprolinemia type II, hypothyroidism, and inflammatory diseases.

Recently, Alonezi et al. used a combination of cisplatin and melittin (a major component of wasp venom) to induce significant changes in cell lines involved in the reduction of metabolite levels during the TCA cycle, oxidative phosphorylation, purine and pyrimidine metabolism, and the arginine/proline pathway [21].

Pathway analysis and enrichment analysis of the first and second categories revealed metabolites associated with thiamine metabolism. Beriberi (as well as Wernicke–Korsakoff syndrome) is caused by a deficiency of thiamine (vitamin B1) [4]. There are two main types: wet beriberi and dry beriberi [20]. Wet beriberi results in a fast heart rate, shortness of breath, and leg swelling. Dry beriberi results in numbness of the hands and feet, confusion, difficulty moving the legs, and pain. A form with loss of appetite and constipation may also occur [20]. Some symptoms of thiamine deficiency are similar to those that can accompany wasp stings, including increased heart rate, shortness of breath, leg swelling, difficulty moving the legs, and pain. Consideration of these similarities and research stemming from this knowledge might inform the development of therapeutic and preventive interventions against the severe sequelae of wasp stings, potentially minimizing the associated morbidity and mortality.

The metabolite concentrations in the third and fourth categories gradually reverted to normal levels with time. Patients with severe symptoms secondary to wasp stings are generally treated by hemodialysis and recover within 72 hours. Zhao et al. studied the recovery of rats after wasp stings and found that all rats recovered after about 24 hours without any treatment [23]. This category of metabolites is relevant to disease symptoms, evaluation of which might be used to assess the recovery of the body and therapeutic effects.

The metabolite levels in the fifth category were not significantly different between the 3-hour group and the control group but were increased in the 24-hour and 72-hour groups. These metabolites may be related to treatment. Pathway analysis and enrichment analysis revealed that these metabolites were associated with the synthesis of catecholamines.

Taken together, our results identified serum metabolites in wasp sting patients and their associated metabolism pathways. Wasp venom is a natural medicine, with complex ingredients and important clinical value that has anti-inflammatory, analgesic, immunomodulatory, microcirculation improving, and tissue repair and recovery properties. However, it is difficult to separate and purify the components of wasp venom. Our study provides important information for understanding the pathogenic mechanisms of wasp sting injuries. The main drawback of this study was its small sample size, which resulted from the rarity of severe wasp sting injuries and complications.

## Data Availability

The data used to support the findings of this study are available from the corresponding author upon request.
